# Consistency of reward responses in neutral and anxious states in adolescent females with anorexia nervosa

**DOI:** 10.1016/j.nicl.2025.103896

**Published:** 2025-10-30

**Authors:** Hayden J. Peel, Nicco Reggente, Michael Strober, Jamie D. Feusner

**Affiliations:** a Centre for Addiction and Mental Health, Toronto, ON, Canada; b La Trobe University, Melbourne, Victoria, Australia,; c Institute for Advanced Consciousness Studies, Santa Monica, CA, United States; d Department of Psychiatry and Biobehavioral Sciences, David Geffen School of Medicine, University of California Los Angeles, Los Angeles, CA, United States; e Department of Psychiatry, Division of Neurosciences & Clinical Translation, University of Toronto, Toronto, ON, Canada; f Department of Women’s and Children’s Health, Karolinska Institutet, Stockholm, Sweden

**Keywords:** Representational similarity analysis, Monetary reward, Anxiety provocation, fMRI, Eating disorders

## Abstract

**Background:**

Anorexia nervosa (AN) is a difficult-to-treat psychiatric disorder that typically onsets in adolescence. AN is marked by atypical reward responsiveness and elevated anxiety. However, the neural dynamics of reward responsivity, and how this is impacted by being in an anxious state, are unknown in AN. To test this, we conducted a novel fMRI task consisting of a monetary reward following anxiety provocation or a neutral condition.

**Methods:**

Forty-seven adolescent participants (25 females with partially or fully weight restored AN, post treatment and 22 mildly anxious controls) were presented with personalized anxiety-provoking or neutral words before receiving a reward. Mildly anxious controls were included to enable dimensional analysis of anxiety severity and to assess whether anxiety effects were specific to AN. To measure trial-by-trial consistency of multivariate patterns associated with reward, and the effects of anxiety on reward, neural responses across reward circuit and cognitive reward control regions were analyzed using representational similarity analysis.

**Results:**

As hypothesized, AN participants showed lower representational similarity than controls during neutral-word rewarded trials, indicating more variable reactivity to rewarding outcomes. Contrary to predictions, there were no significant between-group differences for the effects of anxiety-words on reward representational similarity, and representational similarity did not predict longitudinal symptom change over six months.

**Conclusions:**

The results demonstrate more variable responses to reward receipt in AN compared with controls, but no significant effects of anxiety states on the consistency of reward responses. These results provide insight into the dynamics of reward processing in AN, which has potential implications for planning and guiding reward-focused interventions.

## Highlights

• Lower neural consistency to monetary reward in adolescent females with AN.
• Acute anxiety does not alter reward response consistency in AN vs controls.
• Reward-focused interventions in AN informed by reduced neural consistency.

## 1 Introduction

Anorexia nervosa (AN) is an eating disorder characterized by restrictive food intake, fear of gaining weight, and disturbed body image (Association, 2013), with one of the highest mortality rates in psychiatry (Bulik et al., 2008, Treasure et al., 2010). Anxiety and disturbances in reward processing— e.g., manifesting as reduced positive affect (anhedonia (Dolan et al., 2022), or where typically appetitive stimuli acquiring aversive qualities or vice versa (“reward contamination”) (Keating, 2010, Rye, 2025)—may contribute to the development and maintenance of core AN symptoms. Yet, the way in which these processes interact remain unclear.

Anxiety is common in AN, and often precedes disordered eating (Kaye et al., 2009, Strober et al., 2006, Swinbourne and Touyz, 2007). Compared to healthy controls (HC), those with AN show increased brain activity when presented with anxiety-provoking stimuli in anxiety-related areas—including the medial orbitofrontal cortex (mOFC), insula, anterior cingulate, amygdala-hippocampus, and medial/lateral prefrontal regions (Brooks et al., 2012, Ellison et al., 1998, Horndasch et al., 2018, Joos et al., 2011, Uher et al., 2004).

Individuals with AN also demonstrate reward processing dysregulation, where differences from healthy controls vary depending on different phases of reward processing, including reward anticipation, receipt, and learning (reviewed in (Haynos et al., 2020). Experiences that are typically rewarding to most, such as eating palatable foods (Cowdrey et al., 2013) and perceiving social rewards or physical touch (Cardi et al., 2013, Crucianelli et al., 2021), are attenuated in AN (Haynos et al., 2021). Attenuation for food rewards is notable given their ‘disorder-specific’ relevance, and is reflected in lower activity in reward-related brain areas of the ventral tegmental area (VTA) and striatum (Brooks et al., 2011, Jiang et al., 2019). Social rewards, though less disorder-specific, show associated diminished dorsomedial prefrontal cortex activation (Via et al., 2015). In contrast, monetary rewards—'disorder-nonspecific’ and less directly tied to weight-, food-, or body-related concerns—appear less affected by potentially conditioned aversive responses. Some studies using these stimuli have found no differences from healthy controls in neural responses to reward receipt (Steding et al., 2019), and others have reported heightened dorsolateral prefrontal cortex (dlPFC) activity during reward anticipation (Ehrlich et al., 2015). Taken together, this contrast highlights the importance of considering both reward type and reward phase when studying AN, as different stimuli and task phases may or may not be affected by the illness, and they engage distinct neural mechanisms.

Given that both anxiety and reward systems are disrupted in AN, an important question is how they interact. Anxiety may bias attention and neural resources toward threat, thereby altering the way rewards are processed. Yet, despite converging evidence for alterations in each system, their interplay—and specifically how acute anxiety states affect reward-related neural responses—remains poorly understood. *Trait* anxiety in AN has been linked to increased ventral striatum activity (Bailer et al., 2017) and to altered amygdala-insula coupling during sucrose reward receipt (Frank et al., 2023), suggesting that individual differences in anxiety may shape how affective and interoceptive signals are integrated during reward. However, the impact of acute *state* anxiety on reward has not been studied in AN, representing an important gap that motivates the current study.

In HCs, higher state anxiety impairs reward learning and can lead to overestimation of environmental instability/unpredictability (Hein et al., 2021). Anxiety symptoms are also associated with reduced functional connectivity between the nucleus accumbens (NAcc) and reward processing and cognitive reward control regions (Anderson et al., 2023). Heightened anxiety may reflect a prioritization of neural resources to respond to threat or anxiety, essentially ‘hijacking’ reward responses. While such mechanisms may operate across a range of anxious states, they have not been empirically tested in AN. This population provides a particularly important context in which to investigate them, as the convergence of elevated anxiety and reward dysregulation raises the possibility that anxiety may exert a stronger, or potentially qualitatively different influence on reward processing in AN. This relationship, if demonstrated, may also have clinical relevance, as evidence that anxiety substantially disrupts or alters reward processing in AN would strengthen the rationale for targeting anxiety in clinical interventions.

Given the complexity of interactions between anxiety and reward systems, conventional univariate approaches may be limited in their ability to detect distributed neural patterns, and those underlying dynamic psychological states. Multivariate methods, such as multivoxel pattern analysis (MVPA) and representational similarity analysis (RSA), can reveal these distributed patterns but remain underutilized in AN research. For instance, one MVPA study (Boehm et al., 2021) revealed weight-status-dependent differences in food reward processing in AN, with higher classification accuracy for food stimuli in underweight participants compared to healthy controls, but not in weight-recovered individuals. RSA (Kriegeskorte et al., 2008) quantifies the similarity of multivoxel activation patterns across trials; in this way it can capture both multivariate, distributed representations and trial-by-trial response consistency. High representational similarity (RS) reflects more consistent, coordinated multivariate responses, potentially indexing stable or conditioned patterns of activation from one stimulus to the next (Faul et al., 2020, Visser et al., 2015). Previously, we used RSA to examine neural responses to anxiety-provoking stimuli in AN, finding heightened RS in prefrontal regions, suggesting consistent cognitive control engagement under anxiety (Seiger et al., 2023). Taken together, these studies illustrate the advantages of multivariate approaches in AN research, but also underscore how rarely they have been applied—and, importantly, that reward and anxiety have only been examined separately.

Here, we used RSA to examine monetary reward responses, which provide a disorder-nonspecific probe of reward processing that is less confounded by weight-, food- or body-related aversive conditioning in AN. Specifically, we studied adolescent participants. Adolescence is a critical developmental period characterized, in part, by ongoing maturation of fronto-striatal reward circuitry, and heightened sensitivity to reward and threat cues (Somerville et al., 2010). This stage also coincides with the typical onset of AN (Zipfel, 2015). Thus, examining how anxiety modulates reward-related neural patterns in adolescents with AN provides an opportunity to understand how disruptions in these systems may interact during a key developmental window of vulnerability. Participants completed a task in which monetary reward and non-reward trials were each preceded by either personalized anxiety-provoking or neutral words, allowing us to test how acute anxiety states modulate reward-related neural representations. While prior analyses from this dataset have examined neural responses to anxiety (Seiger et al., 2023) and reward *motivation* network connectivity (Tadayonnejad et al., 2022, Derissen et al., 2023), the current study focuses on reward *receipt*, and how it may be modulated differentially by being in an emotionally neutral or an anxious state.

Our pre-registered hypotheses [https://aspredicted.org/F3D_418] were as follows: 1) We hypothesized that adolescents with AN would show more variable (inconsistent) reward responses (lower RS) during neutral emotional states in reward regions compared to controls, reflecting the behavioural and neural evidence of disrupted reward processing in AN (Brooks et al., 2011, Berridge et al., 2009, Sweitzer et al., 2018). 2) Given the salience of personalized anxiety-inducing stimuli (Joos et al., 2011), and as we previously demonstrated multivariate anxiety responses to be more consistent in AN than controls (Seiger et al., 2023), we hypothesized that this consistent/coordinated response to anxiety provocation in AN may lead to increased consistency (i.e., dampened consistently trial-by-trial, resulting in higher RS) in reward responses within reward regions following anxiety provocation, compared with responses in control participants. 3) We hypothesized that greater similarity of reward-related activity patterns between anxiety- and neutral-cued trials would predict worse clinical course, reflected in decreasing adjusted BMI and increasing eating disorder symptom severity over the six months after intensive treatment. The rationale for this prediction was that individuals with AN who show little differentiation in reward responses across emotional states, reflecting more consistently dampened reward processing, would have poorer longitudinal outcomes. 4) In line with the attenuated subjective and neural reward response in AN, we hypothesized that lower consistency of reward responses in the neutral condition will be predictive of poorer Behavioral Activation System (BAS) reward responsiveness scores, which quantifies subjective responses to reward anticipation and receipt (Carver and White, 1994).

## 2 Materials and methods

### 2.1 Participants

The UCLA Institutional Review Board approved the study. Female adolescents aged 10–19 were recruited from the greater Los Angeles area. Written informed consent was obtained from participants and their guardians for those under 18. Participants with AN met DSM-5 criteria for the restrictive subtype and were partially or fully weight-restored, post-treatment. Non-clinical, controls were free from DSM-5 diagnoses but scored above population norms on the anxiety subscale of the DASS-21. (This group with mild anxiety was included in the interest of planned dimensional analyses of anxiety across all participants as part of the larger study and as previously reported (Seiger et al., 2023). (See Supplementary Materials S1 for details on recruitment, full inclusion and exclusion criteria.)

Clinical evaluations for all participants were conducted by licensed psychiatrists or psychologists experienced with this population. Primary or comorbid diagnoses were screened using the Mini-International Neuropsychiatric Interview (MINI KID 7.0.2) (Sheehan et al., 2010). To measure depression severity, the Children's Depression Rating Scale™, Revised (CDRS™-R) (Overholser et al., 1995) and the depression subscale of the Depression Anxiety Stress Scale (DASS-21) (Lovibond and Lovibond, 1995) were administered. We also administered the Behavioral Inhibition System/Behavioral Activation System (BIS/BAS) (Carver and White, 1994) questionnaire (for this analysis we focused on the BAS Reward Responsiveness scale, as the construct it measures most closely corresponds to the responsivity to reward portion of the fMRI task). The Hamilton Anxiety Rating Scale (HAMA) (Hamilton, 1959) measured past week anxiety. Additionally, participants rated their level of anxiety immediately after scanning on a Likert scale from 0 to 10. To evaluate the severity of AN symptoms, the Eating Disorder Examination (EDE) and the Yale-Brown-Cornell Eating Disorder Scale (YBC-EDS) (Jordan et al., 2009) were administered. The study was registered on Clinicaltrials.gov NCT02948452.

### 2.2 fMRI task paradigm

**Fig. 1:**
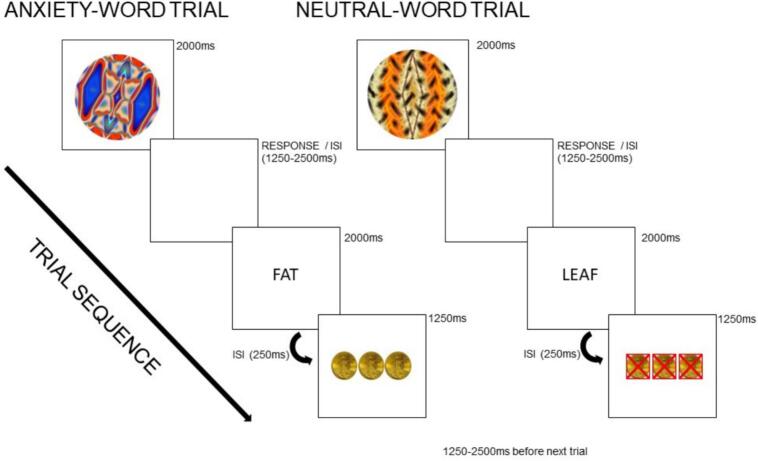
Schematic of anxiety-word and neutral-word reward trials. Each trial began with a fractal image followed by an individually-tailored anxiety-provoking or neutral word, then a response period in which the participant guessed if the image belonged in “Group 1 or Group 2,” then visual feedback of coins (or red Xs over coins) indicating if they received reward or non-reward, respectively. *ISI* = inter-stimulus interval. (For interpretation of the references to colour in this figure legend, the reader is referred to the web version of this article.)

Participants completed a fast event-related fMRI paradigm involving a monetary reward classification task at two time points, two days apart, with the task order randomized. Each scanning session used a distinct set of six fractals, selected from a pool of twelve. At the beginning of each trial, one of six colored fractal images was presented for 2000 ms. Fractals were arbitrarily assigned and counterbalanced across participants to ensure that each set was unique to a given session. Participants guessed whether it belonged to “Group 1” or “Group 2” by pressing a button, followed by a jittered inter-stimulus interval (ISI; 1250–2500 ms). Optimized jittering was calculated with optseq2 (https://surfer.nmr.mgh.harvard.edu/optseq). Next, a word stimulus—either neutral or anxiety-provoking—was displayed for 2000 ms, depending on the session. Another brief ISI (250 ms) preceded feedback indicating whether they had received a $10 reward. Rewards were randomly assigned with a 50 % probability independent of participant response, which eliminated the potential for learning effects since the goal of the study was to examine responses to reward receipt. To reduce frustration, participants were told that sometimes a correct answer resulted in the wrong feedback, and vice versa. Trials concluded with another jittered ISI (1250–2500 ms) before the next fractal was presented. In total, 60 trials were administered per session, evenly distributed across four conditions: rewarded and non-rewarded trials following anxiety or neutral words. See Fig. 1 for a visual depiction of the task.

Individually-tailored word stimuli were chosen based on a pre-scan word rating task, where participants rated 150 words on a 1–9 Likert scale of “how anxious you feel when you think about the meaning of these words.” There were 50 disorder-related, potentially anxiety-provoking words (e.g., diet, weight), 50 generally anxiety-provoking words (e.g., intimidate, pain), and 50 neutral words (e.g., leaf, bus). For the AN group, the 20 highest-rated anorexia-related words were used in the anxiety condition; for controls, the top-rated general anxiety words were used. Neutral stimuli consisted of the 20 lowest-rated neutral words, with syllable count matched across word sets. Participants were randomly assigned to the anxiety or neutral session first. The presentation of anxiety-related versus neutral words was independent of reward outcomes, and post-scan ratings confirmed that the anxiety-word condition increased subjective anxiety (see Results section 3.2).

### 2.3 MRI data acquisition and processing

MRI data were acquired on a 3 T Siemens PRISMA scanner with a 64-channel coil. Echoplanar images (EPI) were collected with the following parameters: repetition time (TR) of 1000 ms, echo time (TE) of 33 ms, flip angle of 80°, isotropic voxel size of 2 mm^3^, multiband acceleration factor of 5, field of view of 208 mm, 487 volumes, and 60 slices. Structural MRI for registration purposes utilized a T1-weighted MPRAGE sequence with a TR of 2300 ms, TE of 2.99 ms, and isotropic voxel dimensions of 0.8 mm^3^.

Functional MRI data preprocessing was done with FSL (FMRIB’s Software Library, https://www.fmrib.ox.ac.uk/fsl) using FEAT version 6.0 (FMRI Expert Analysis Tool). This included motion correction (MCFLIRT) and temporal filtering. The unsmoothed, native functional space fMRI runs from each participant were then forwarded to the RSA preprocessing pipeline (see 2.5 below). Additionally, T1-weighted scans were brain-extracted using BET and used for standard space registration with FLIRT before conducting RSA.

### 2.4 Regions of interest

Core areas involved in reward comprised the reward region of interest (ROI) mask, including bilateral basolateral amygdala, VTA, NAcc, mOFC, as well as “cognitive reward control” regions – dlPFC, left ventrolateral prefrontal cortex (vlPFC), and supplementary motor area (SMA) − from a previous coordinate-based *meta*-analysis of fMRI tasks in which participant cognitively controlled hedonic impulses towards rewarding cues (Brandl et al., 2019). Probabilistic atlases thresholded at 50 % were used to extract bilateral basolateral amygdala (Harvard-Oxford), mOFC (aal2) and SMA (aal2). To create masks corresponding to cognitive reward control, the left vlPFC mask was created based on the Harvard-Oxford atlas for the pars triangularis, thresholded at 50 %. The bilateral dlPFC was created by first merging the superior- and middle-frontal probabilistic maps from the Harvard-Oxford atlas, then, the voxels overlapping these merged maps and the association map generated from the Neurosynth fMRI *meta*-analytic database (neurosynth.org) using the search term “DLPFC” were used as the final mask. The Pauli and colleagues (Pauli et al., 2018) probabilistic atlas was used for the VTA and NAcc, but given these are small, a liberal probability threshold of p > 0.001 was applied, meaning that voxels with at least 0.1 % probability of belonging to the region were included in the mask. All regions were then merged into a single ROI mask.

### 2.5 Representational similarity analysis (RSA)

We conducted RSA within the ROIs using custom MATLAB scripts, generating voxel-wise RSA maps for each participant across four reward periods (∼30 trials each): anxiety-word rewarded, neutral-word rewarded, anxiety-word non-rewarded, and neutral-word non-rewarded trials. We used the same protocol for calculating beta maps as in our previous RSA study (Seiger et al., 2023). Briefly, we extracted the relevant time series corresponding to the reward and non-reward period of the anxiety and neutral runs using custom MATLAB scripts. The preprocessed time series data in native space were then forwarded to Analysis of Functional Neuroimages (AFNI) (Cox, 1996) and deconvolved using the 3dDeconvolve command. We conducted least-square-sum estimates of beta values using 3dLSS for each reward period within the respective run. The ∼30 single-trial beta maps from each participant for the reward periods were transformed into MNI standard space using the previously calculated transformation matrices within FSL and then forwarded to the RSA pipeline. Time points with movement outliers were identified using FSL's motion outlier tool (fsl_motion_outliers) with the DVARS metric (Power et al., 2012) and default threshold (75th percentile + 1.5 × interquartile range of DVARS values within each run). Across participants, the number of excluded volumes ranged from 0 to 65 per run (mean ≈ 12), corresponding to approximately 0–13 % of the 487 volumes acquired per run.

Each RSA consisted of an *N* × *N* similarity matrix, where *N* is the total number of trials (∼30) for each word/reward-combination, with each element of the matrix representing the correlation between voxel patterns in the ROIs for the trials. These similarity matrices were generated by correlating the single-trial beta maps from the different trial types. We also pre-registered (https://aspredicted.org/F3D_418) an exploratory analysis to test whether the reward state influences the subsequent anxiety state (Supplementary Materials S3). A searchlight mapping approach (Kriegeskorte et al., 2006) within the ROI masks was also used, creating spherical ROIs with a 2-voxel radius (∼30–33 voxels per sphere) centered on each voxel. Searchlights intersecting mask boundaries necessarily contained fewer voxels but typically retained the majority. We calculated the mean Pearson correlation across all trials within each condition for each spherical ROI. This resulted in similarity maps for each period for each participant, where each voxel's value represented the average correlation (*r*-value) across all trials when the spherical ROI was centered on that voxel. Scripts used to conduct these analyses are freely available: https://github.com/Institute-for-Advanced-Consciousness/Anorexia-Reward-RSA.

### 2.6 Statistical analyses

Statistical analyses were preregistered (https://aspredicted.org/F3D_418https://aspredicted.org/F3D_418), with separate analyses for the neutral and anxiety-reward conditions. This approach was guided by both theoretical and statistical considerations. Namely, we aimed to characterize how reward-related neural patterns differ by group *within* distinct emotional states, rather than test for an interaction between condition and group. Statistically, a more complex Group × Condition model (from analyzing both word conditions in a single omnibus ANOVA) would have required greater power to detect interaction effects, which is difficult given a modest sample (*N* = 47). By modeling each condition separately, we maintained statistical sensitivity to detect group differences within each state, consistent with our preregistered hypotheses. Power analysis with G*Power indicated that separate ANCOVAs were adequately powered (α = 0.05, 1 – β = 0.80) to detect medium-to-large effects (f ≈ 0.42).

### 2.7 Group analyses

#### 2.7.1 Averaged ROI analysis

To compare multivariate responses across the reward mask, we r-to-z transformed and averaged the RS values, yielding a single z-value per participant for each condition and ROI (Dimsdale-Zucker and Ranganath, 2018). After log-transformation due to non-normality, these values were analyzed using ANCOVAs with group as the factor, pubertal development scale (PDS) scores as a covariate, and *p* < 0.05, two-tailed, as the statistical threshold. Although we also planned to use age as a covariate per our pre-registered hypotheses, there was high collinearity between age and PDS; thus we chose only to include PDS (total score range 5–20, with higher scores reflecting more advanced pubertal development) as it better represents developmental stage, particularly in AN populations previously affected by starvation. Second, as specified in the preregistration, we carried out a sensitivity analysis to assess whether the mask-level effect was disproportionately influenced by any single ROI. For this, we conducted a series of leave-one-ROI-out MANOVAs with Group as a factor and PDS as a covariate, in which one ROI was iteratively omitted from the set of multivariate dependent variables.

#### 2.7.2 Searchlight analysis

Within the ROI, group differences were assessed with FSL Randomise, including PDS values as a covariate of non-interest. We conducted a non-parametric permutation test with 5000 permutations and a threshold-free cluster enhancement method (Smith and Nichols, 2009), applying a statistical threshold of *p* < 0.05, FWE-corrected. As post hoc analyses, within-group responses for different conditions were tested using one-sample t-tests with PDS as a covariate.

### 2.8 Associations with clinical variables

We conducted linear mixed models (LMM, unstructured covariance structure for random effects) on data from the AN group, with PDS as a covariate. The first model included adjusted-BMI (i.e., BMI-percentiles) as the dependent variable, with time (adjusted-BMI entry, and monthly intervals to 6-month follow-up) and 'across anxiety and neutral reward' RS as factors. This RS was calculated by computing all pairwise correlations between multivoxel activation patterns from reward trials preceded by anxiety-provoking words with multivoxel activation patterns from reward trials preceded by neutral words. Correlations were then Fisher z-transformed and averaged across the ROI mask. With this value, higher RS values indicate more consistent reward representations across contexts, whereas lower values indicate greater differentiation. The second LMM was similar but used eating disorder examination (EDE) global scores as the dependent variable and included only study entry and 6-month follow-up time points. The final LMM used BAS as the dependent variable, with time (BAS entry and 6-month follow-up) and RS values from the neutral-word reward period as factors (without an interaction term).

Post hoc exploratory Pearson’s and Spearman’s partial correlations (with PDS as a covariate) were used to examine baseline associations between symptom severity measures and RS that differed between groups in the ANCOVA. We applied the Benjamini-Hochberg False Discovery Rate (FDR) correction to these *p*-values.

## 3 Results

### 3.1 Sample characteristics

Twenty-five females with AN (14.6 ± 1.9 years) and 22 control participants (16.2 ± 1.8) were included in analyses. Several participants were excluded due to excessive motion (*n* = 3), errors with the task (*n* = 7), missing or incomplete data (*n* = 7), and binge-purge subtype (*n* = 2). Demographic and psychometric information are shown in Table 1.

**Table 1:** Demographics and psychometrics.

	AN (*N* = 25)	CON (*N* = 22)	Between-group statistics		
			*W*	*t*	*p*-value
Age in Months (M ± SD)	175.36 (23.54)	194.73 (21.25)		−2.94	0.005
Pubertal Development Scale (M ± SD)	14.16 (4.33)	17.86 (1.42)	111.00		< 0.001
					
Adjusted-BMI (percentile) (M ± SD)	27.28 (17.81)	63.00 (23.07)		−5.98	< 0.001
Symptom Severity (M ± SD)					
HAMA-A	14.28 (7.80)	7.23 (4.49)		3.73	0<.001
EDE	3.50 (1.63)	0.53 (0.74)	36.50		0<.001
BAS Reward Responsiveness	16.54 (2.21)	17.27 (2.10)		−1.14	0.257
YBC-EDS	24.38 (10.92)	1.50 (3.16)	19.00		< 0.001
DASS-depression	19.91 (10.64)	9.43 (6.87)		3.84	< 0.001
CDRS	42.25 (18.56)	23.09 (5.48)	58.00		< 0.001
DASS-anxiety	14.17 (9.64)	14.10 (6.21)		0.29	0.977
Psychiatric Comorbidities^a^					
Major depressive episode	8	−			
Obsessive compulsive disorder	5	−			
Panic disorder	3	−			
Agoraphobia	1	−			
Specific phobia	1	−			
Generalized social phobia	4	−			
Generalized anxiety disorder	12	−			
Body dysmorphic disorder	1	−			
Separation anxiety disorder	2	−			
No DSM comorbid disorder	7	−			
Medication	18	0			

*AN* anorexia nervosa, *CON* controls, *BMI* body mass index, *HAM-A* Hamilton Anxiety Rating Scale, *EDE* Eating Disorder Examination, *BAS* Behavioral Activation Scale*, YBC-EDS* Yale-Brown-Cornell Eating Disorder Scale, *DASS* Depression Anxiety Stress Scales, *CDRS* Children’s Depression Rating Scale, *t* t-statistic, *W* Mann-Whitney U statistic.

a Note: some AN participants had more than one comorbid disorder.

### 3.2 Subjective task-related anxiety

Anxiety ratings post-scan were significantly higher in the AN group than the controls following the anxiety-word condition (AN = 5.38 ± 2.32; HC = 2.55 ± 1.77; *p* < 0.001). Anxiety ratings were also collected after neutral runs (AN = 4.80 ± 2.43; HC = 2.23 ± 2.11). However, ratings in the control group were heavily zero-inflated, and the non-zero data points had a narrow range. While alternative approaches (e.g., zero-inflated models) can address such distributions, these data were not further analyzed given their limited range and peripheral relevance to the study aims.

### 3.3 Between- and within-group results

ANCOVA revealed a significant difference between groups for the neutral-word rewarded trials (*F*_(1, 44)_ = 6.44, *p* = 0.015, η^2^ = 0.128), driven by lower RS in the AN group compared to controls in the ROI mask. (For completeness, the same analysis for non-rewarded trials was non-significant: *F*_(1, 44)_ = 0.97, *p* = 0.333, η^2^ = 0.023; see Supplementary Materials S2). Iteratively removing each region from the reward ROI mask and performing leave-one-ROI-out MANOVA did not reveal significant differences in any model (all *p* > 0.148), suggesting that the between-group mask-level results were not heavily influenced by any one region within the overall reward mask.

**Fig. 2:**
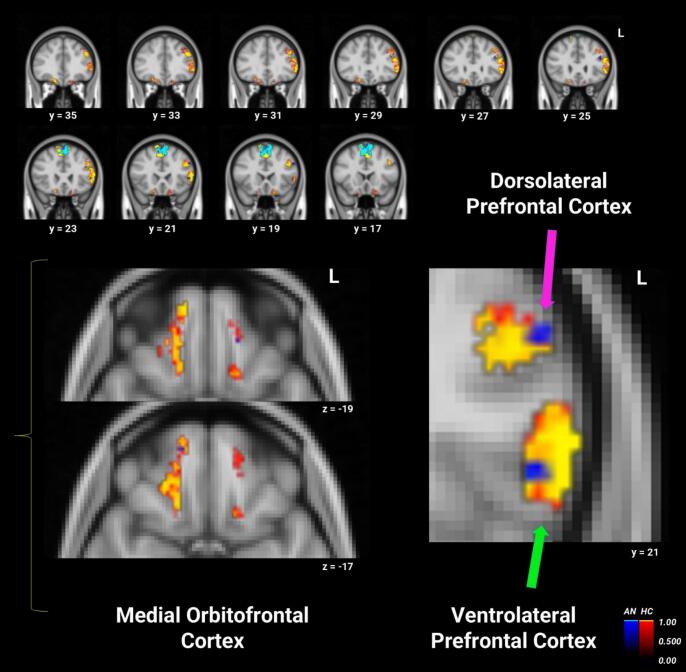
Within-groups one sample *t*-tests for neutral-word rewarded trials’ representational similarity, from the searchlight analysis within the mask that included reward and cognitive reward control regions. Results for healthy controls (red/yellow) and anorexia nervosa (AN; blue/cyan) are overlaid (*p* < 0.05, TFCE- and FWE-corrected). Colors represent group-specific statistical significance (1 – *p*)*,* not activation magnitude or direction. See Table 2 for details for each cluster. (For interpretation of the references to colour in this figure legend, the reader is referred to the web version of this article.)

Between-group searchlight analyses within the ROI mask did not reveal significant differences between groups. However, within-group results showed significant clusters for the control group which had (qualitatively) larger spatial extents or were not significant in the AN group. Subcortical reward circuit areas not significant in AN included the right NAcc and left basolateral amygdala, as well as left mOFC. Cognitive reward control areas that were significant in both groups and (qualitatively) had a similar magnitude but a smaller spatial extent (i.e., voxel size) in AN included left vlPFC and left dlPFC, as well as right mOFC (see Fig. 2 and Table 2).

**Table 2:** Searchlight analysis within-group one-sample *t* – test for neutral-word rewarded trials for AN and controls.

Region	Size (voxels)	T max	Cluster #	MNI Coordinates		
				*X*	*Y*	*Z*
Control Group						
Supplementary Motor Area	2862	8.62	8	10	−16	72
L vlPFC/dlPFC	806	6.58	7	−50	28	22
R mOFC	232	6.75	6	16	36	–22
L mOFC	65	5.97	5	−16	48	−18
L mOFC	44	6.90	4	−16	20	−20
L Basolateral Amygdala	2	6.95	3	−26	−4	−26
R NAcc	2	8.66	2	6	8	−8
AN Group						
Supplementary Motor Area	2122	8.22	7	8	−10	52
L dlPFC	33	6.42	6	−40	28	24
L vlPFC	23	6.03	5	−48	22	4
L dlPFC	19	5.61	4	−50	20	32
R mOFC	4	6.61	3	14	54	−16
L dlPFC	4	4.67	2	−44	22	32

*AN* anorexia nervosa, *L* left hemisphere, *R* right hemisphere, *vlPFC* ventrolateral prefrontal cortex, *dlPFC* dorsolateral prefrontal cortex, *mOFC* medial orbitofrontal cortex, *NAcc* nucleus accumbens, *MNI* Montreal Neurological Institute.

**Fig. 3:**
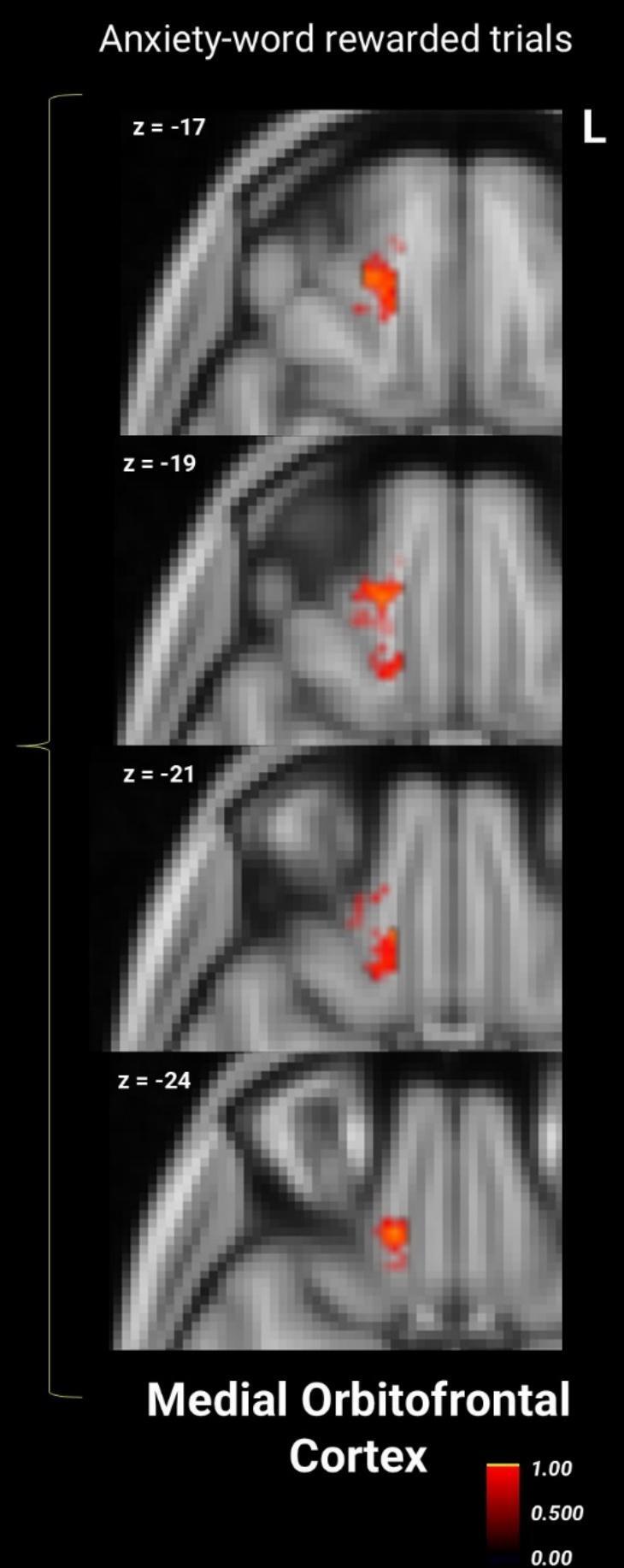
Within-group one-sample *t*-tests for the anxiety-word rewarded trials representational similarity from the searchlight analysis within the reward mask. Within-group results are overlaid: red/yellow clusters depict significant areas in controls, while there are no significant blue/cyan clusters in AN (*p* < 0.05, TFCE- and FWE-corrected). Colors represent group-specific statistical significance (1 – *p*)*,* not activation magnitude or direction. (For interpretation of the references to colour in this figure legend, the reader is referred to the web version of this article.)

ANCOVA found no significant group differences in RS during anxiety-word rewarded trials (*F*_(1, 44)_ = 1.72, *p* = 0.197, η^2^ = 0.038). (For completeness, the same analysis for anxiety-word non-rewarded trials was also non-significant: *F*_(1, 44)_ = 0.39, *p* = 0.536, η^2^ = 0.009; see Supplementary Materials S3). Similarly, the between-group searchlight analyses did not reveal any significant differences between groups, and MANOVA did not find that any one region contributed more strongly to results. However, exploratory within-group searchlights revealed that controls had significant RS clusters in the left mOFC and left vlPFC that were not significant in AN (Fig. 3, Table 3).

**Table 3:** Searchlight within-group one-sample *t* – test for anxiety-word rewarded trials for AN and controls.

Region	Size (voxels)	T max	Cluster #	MNI Coordinates		
				*X*	*Y*	*Z*
Control Group						
Supplementary Motor Area	2808	7.71	4	14	10	64
L vlPFC	369	6.83	3	−54	30	2
L dlPFC	251	6.87	2	−42	20	34
BL mOFC	110	6.91	1	14	30	−24
AN Group						
Supplementary Motor Area	2799	8.24	3	−6	−6	66
L dlPFC	361	7.61	2	−48	28	28
R mOFC	1	6.06	1	16	36	−20

*AN* anorexia nervosa, *L* left hemisphere, *R* right hemisphere, *BL* bilateral, *vlPFC* ventrolateral prefrontal cortex, *dlPFC* dorsolateral prefrontal cortex, *mOFC* medial orbitofrontal cortex, *MNI* Montreal Neurological Institute.

### 3.4 Associations with clinical variables

There was no main effect of time (all *p* > 0.341), RS (*p* = 0.183), or their interaction (all *p* > 0.709) on adjusted-BMI. Similarly, with EDE as the dependent variable, no significant effects of time (*p* = 0.088), RS (*p* = 0.486), or their interaction (*p* = 0.693) were detected. Lastly, there was no main effects for time (*p* = 0.631) or RS (*p* = 0.103) on BAS scores. In summary, the difference in RS between anxiety and neutral reward trials did not predict longitudinal adjusted-BMI or EDE changes, and neutral-word rewarded trial RS were not associated with BAS scores.

Exploratory Pearson and Spearman's ρ partial correlation analyses examined the relationship between neutral-word rewarded trial RS and clinical measures at baseline, with PDS as a covariate. Across the entire sample, a negative relationship between RS and CDRS was observed (ρ = -0.33, *p* = 0.028, FDR adjusted *p* = 0.084) (not surviving correction for multiple comparisons). However, because CDRS is collinear with diagnostic group, we examined correlations separately within groups. These associations were weaker and nonsignificant (AN: ρ = –.06, *p* = 0.77; HC: ρ = –.28, *p* = 0.21), suggesting that the across-sample trend may have been driven by mean group differences in CDRS rather than a consistent within-group relationship. No significant relationship was found between RS and HAM-A (ρ = -0.14, *p* = 0.345, FDR adjusted *p* = 0.345), or adjusted-BMI (ρ = 0.24, *p* = 0.098, FDR adjusted *p* = 0.147). In the AN group, no significant relationships were detected between RS and EDE (*r* = 0.05, *p* = 0.811) or YBC-EDS (*r* = 0.21, *p* = 0.326).

## 4 Discussion

This study examined the neural consistency associated with receipt of monetary reward, and how it is affected by being in an anxious state, in adolescent females with AN and non-clinical controls. As hypothesized, AN participants showed lower consistency (lower RS) in reward-related regions during neutral-states. Contrary to predictions, anxiety provocation did not differentially modulate reward processing between groups. Similarly, the difference in neural consistency between anxiety and neutral reward trials did not predict longitudinal adjusted-BMI or eating disorder symptom improvement, and subjective reward responsiveness was not associated with neutral-word reward neural consistency.

The primary finding of the study was that adolescents with AN have reduced neural consistency during the neutral-state when receiving rewarding stimuli, compared with controls. The absence of group differences for the neutral-word non-rewarded trials suggests that observed effects are specific to reward receipt, rather than reflecting a general difference in trial-to-trial neural consistency. This finding extends previous univariate neuroimaging studies that have reported attenuated neural responses to monetary reward in AN (Decker et al., 2015, Wagner et al., 2007, Wierenga et al., 2015), raising the possibility that reward-related signals in AN may also be less reliably represented across trials. One potential mechanism is less consistent hedonic engagement with monetary rewards in AN, consistent with neurobiological models that implicate limbic and frontostriatal circuits in reward “liking” (Berridge and Kringelbach, 2015, Walsh, 2013). While these models do not explicitly address trial-to-trial neural consistency, our findings suggest that individuals with AN may engage these systems more variably across repeated exposures. Alternatively, excessive self-regulation in AN, particularly if it is inconsistent, may lead to variable reward-related activity. Excessive self-regulation is supported by cognitive-behavioral models of AN (Fairburn et al., 1999), and broader neurocognitive frameworks describe how prefrontal control systems modulate reward regions (Kober et al., 2010, Miller et al., 2001). Applying these models to AN suggests that altered or dysregulated top-down control may disrupt stable reward-related representations.

Although between-group searchlight differences were not significant for the neutral-state reward trials, exploratory within-group analyses identified RS clusters in prefrontal regions—including the dlPFC, vlPFC, and mOFC—in both groups, though the spatial extent of these clusters was notably larger in controls than in AN. Subcortical RS clusters in the right NAcc and left basolateral amygdala were only observed in controls. While these within-group patterns do not establish regional group differences, their overall topography—widespread RS in controls versus more spatially limited or not significant in AN—offers qualitative support for the group-level difference observed in the ANCOVA described above. That is, the spatial distribution of RS is consistent with the idea that reward representations are more stable and widely distributed in controls, but may be disrupted in AN. Nonetheless, these interpretations should be considered hypothesis-generating for future studies, as they are not based on statistically significant between-groups searchlight effects.

Contrary to our hypothesis, state anxiety did not increase neural consistency in reward regions for individuals with AN compared to controls. While between-group differences during anxiety-word reward trials were not statistically significant, exploratory within-group analyses revealed statistically consistent RS clusters in the left vlPFC and mOFC in controls, but not in AN. This raises the possibility of diminished consistency in cognitive reward control and reward circuitry responses in AN. Put more plainly, healthy controls reliably engaged prefrontal regions when anxious, trial-by-trial, whereas this pattern was absent in AN. This may suggest less stable recruitment of these systems in AN, but such an interpretation must be regarded as preliminary and hypothesis-generating given the absence of significant between-group effects. The fact that the non-clinical controls had mild anxiety may have contributed to non-significant between-group differences. While this sampling strategy was intended to facilitate dimensional phenotypic analyses with anxiety severity across the sample (as previously reported (Seiger et al., 2023), it may have limited our ability to detect anxiety-related modulation differences at the group level.

None of the preregistered brain-behaviour associations were supported. The across-context similarity of reward-related activity patterns (anxiety vs. neutral) were not associated with the longitudinal clinical outcomes of changes in weight-related or eating disorder symptoms, suggesting these RS patterns may not serve as indicators of symptom change. We used monetary reward as it may be a ‘disorder-irrelevant’ stimulus—less likely to evoke conditioned aversive responses than food or body-related stimuli—to probe general reward processing in AN. This approach allowed us to examine neural consistency in response to reward cues without the confounds of disorder-specific emotional salience. However, the lack of association with clinical outcomes suggests that general reward responsiveness, while informative about underlying neural mechanisms, may not directly map onto symptom change. Similarly, neural consistency during neutral-state reward was not associated with self-reported reward sensitivity and approach motivation (BAS scores) at baseline or follow-up, and importantly, BAS scores did not significantly differ between groups. This suggests that reduced neural consistency in AN may reflect more subtle or dynamic aspects of reward processing—such as trial-to-trial variability or interference from regulatory processes—that are not well captured by trait-based self-report measures. Further, an independent analysis of a different task phase in the same sample found that functional connectivity in reward circuits also failed to predict longitudinal clinical outcomes (Visser et al., 2015), providing converging evidence the core measures of reward circuitry function may not align with symptom trajectories. Lastly, exploratory correlations showed no significant associations between baseline eating disorder severity (EDE or YBC-EDS scores) and neural consistency. Taken together, these findings suggest that clinical and self-report measures may not be tightly coupled with neural indices of general reward responsiveness in AN. However, future research may benefit from examining neural consistency in response to disorder-relevant stimuli, to clarify how representational similarity in those contexts relates to clinical outcomes.

Although preregistered associations with clinical outcomes were not supported, exploratory correlations found trend-level, medium effect size relationships between worse depressive symptoms and lower reward system neural consistency across the sample. However, this effect was weaker within each of the AN and HC groups, suggesting it may have been driven primarily by mean differences in depressive symptoms between AN and controls. Given that depressive symptom of anhedonia (the loss of pleasure in previously rewarding stimuli) has been found in a *meta*-analysis to be higher in eating disorder populations than controls (Dolan et al., 2022), and is related to dysregulated reward processing (for a review, see (Heshmati and Russo, 2015), it remains possible that depression-related processes contribute to the broader group-level differences in neural consistency, although without knowledge of patterns in those with intermediate-level depression, a dimensional association across individuals cannot be concluded. Finally, not only were there no significant group differences in reward response consistency in anxiety-induced states, there were also no significant relationships between reward response consistency and anxiety symptoms (HAM-A). These results therefore do not provide evidence of a differentially strong interplay between anxiety and reward in AN, at least in terms of consistency of neural responses.

Lower reward response consistency in adolescents with AN compared to controls may have clinical implications. It is possible that this might contribute to their diminished ability, as shown in previous studies, to derive pleasure from money (Decker et al., 2015), social engagement (Hartmann et al., 2010), and food (Cowdrey et al., 2013) rewards. Without reliable experiences of pleasure from these stimuli, those with AN may not readily seek out opportunities to engage with these. Positive Affect Treatment approaches for AN (Haynos et al., 2021), a cognitive-behavioral intervention targeting reward deficits associated with anticipation, experiencing, and learning, may therefore benefit from incorporating neural consistency measures like RS to monitor intervention effects, perhaps either neurally or with identifying proxy physiological measures that are sensitive to consistency of responses. To establish and validate this approach, this paradigm and analyses would need to be incorporated into mechanistic clinical trials to understand if the neural reward receipt can be used as a biomarker of response.

This study has several limitations. The sample size may have limited our power to detect group differences, particularly for the searchlight analyses. It also limited our ability to conduct sub-analyses of medicated and un-medicated AN groups and to account for comorbidities; while correlation analyses with anxiety and depressive symptoms probed for specific dimensional relationships, they may not fully account for all the effects of comorbid disorders. Replicating this study with a larger sample could help address these issues. Additionally, the study population was at a specific stage of illness recovery—immediately after weight-restoring intensive treatment—, included only females, and focused on the restrictive subtype of AN; therefore, the results might not generalize to other illness stages, males, or those with binge-purge subtype presentations. In particular, potential sex differences in brain development and function could lead to different patterns of reward–anxiety interactions (Somerville et al., 2010), highlighting the need for future research in more diverse samples.

In conclusion, this study demonstrates lower neural consistency during reward receipt in adolescents with AN compared to mildly anxious controls. Potentially accounting for this is low consistency of responses in AN in core nodes of the reward circuit, and in cognitive reward control regions. However, induced anxiety-states did not affect reward responses differentially in AN and controls. The results from these analyses have implications for future research and for the ongoing development of treatment approaches targeted towards improving reward processing in AN.

## CRediT authorship contribution statement

**Hayden J. Peel:** Writing -- original draft, Writing -- review & editing, Formal analysis. **Nicco Reggente:** Writing – review & editing, Formal analysis. **Michael Strober:** Writing – review & editing, Methodology, Conceptualization. **Jamie D. Feusner:** Writing – review & editing, Writing – original draft, Methodology, Funding acquisition, Formal analysis, Conceptualization.

## Declaration of competing interest

The authors declare that they have no known competing financial interests or personal relationships that could have appeared to influence the work reported in this paper.

## Data Availability

The data that support the findings of this study are openly available in the NIMH Data Archive at https://nda.nih.gov/, collection ID 2565.
