# Supplementary material for: Consistency of reward responses in neutral and anxious states in adolescent females with anorexia nervosa
Source: Neuroimage Clin. 2025 Oct 30;48:103896. doi: 10.1016/j.nicl.2025.103896 (PMC12634276; doi:10.1016/j.nicl.2025.103896)
Supplement: Supplementary Data 1 [file mmc1.docx]

**Supplementary Material**

**Title**

Consistency of reward responses in neutral and anxious states in adolescent females with anorexia nervosa

**Authors**

Hayden J. Peel^1, 2^, Nicco Reggente^3^, Michael Strober^4^, Jamie D. Feusner^1,4,5,6^

^1^Centre for Addiction and Mental Health, Toronto, ON, Canada

^2^La Trobe University, Melbourne, VIC, Australia

^3^Institute for Advanced Consciousness Studies, Santa Monica, CA, United States

^4^Department of Psychiatry and Biobehavioral Sciences, David Geffen School of Medicine, University of California Los Angeles, Los Angeles, CA, United States

^5^Department of Psychiatry, Division of Neurosciences & Clinical Translation, University of Toronto, Toronto, ON, Canada

^6^Department of Women’s and Children’s Health, Karolinska Institutet, Stockholm, Sweden

**Contents:**

**Supplementary Methods**

1. Recruitment and inclusion / exclusion criteria
2. Details on non-reward trial analyses
3. Details on exploratory anxiety-word post-reward analysis

**Supplementary Tables**

**Table S1** Analysis of covariance for neutral-word rewarded trials RSA with PDS as a covariate

**Table S2** Searchlight analysis within-group one-sample t-test results for anxiety-word non-rewarded trials

**Table S3** Searchlight analysis within-group one-sample t-test results for neutral-word non-rewarded trials

**Table S4** Searchlight analysis within-group one-sample t-test results for anxiety-word post-rewarded trials

**Supplementary Figures**

**Figure S1** Within-group one-sample t-test results from searchlight analysis within the reward mask for the anxiety-word and neutral-word non-rewarded trials

**Figure S2** Within-group one-sample t-test results from searchlight analysis within the anxiety mask for the anxiety-word post-rewarded trials

**Supplementary References**

1. **Recruitment and inclusion / exclusion criteria**

AN participants were recruited from the UCLA inpatient eating disorder unit and local treatment centers, enrolled at the end of their treatment once meeting criteria for lower level of care, and were partially or fully weight-restored. Participants with AN met the DSM-5 criteria for the restricting type of AN. Inclusion criteria for the AN group were: Ages 10–19; met DSM-5 criteria for Anorexia Nervosa, Restricting Type, within the previous 6 months; completed usual treatment in an inpatient, residential, or partial hospitalization program (2–5 times/week) involving psychotherapy and dietary monitoring within the previous 3 weeks; were either un-medicated or taking a stable dose of serotonin reuptake inhibitors for at least 8 weeks; and finally, they could not be on any other psychotropic medications except for a short half-life sedative/hypnotic for insomnia or a short half-life benzodiazepine for anxiety, not exceeding three doses per week and not taken on scan days. Exclusion criteria for the AN group included a lifetime history of bipolar disorder, psychotic disorders, attention-deficit hyperactivity disorder, or current post-traumatic stress disorder.

Controls were recruited via online and community advertisements. Inclusion criteria for control participants were females aged 10–19 who scored at least 0.5 standard deviations higher than population norms on the anxiety portion of the Depression Anxiety Stress Scale (DASS-21). This helped us better examine the relationship between anxiety and brain function and structure across AN and control groups. Exclusion criteria for control participants were any DSM-5 diagnosis, assessed with the MINI KID 7.0.2, and use of any psychiatric medication.

Exclusion criteria for all participants were: current substance abuse or dependence, including nicotine; pathological gambling, as assessed with the South Oaks Gambling Screen; current medical and neurological disorders (e.g., diabetes, hypertension, seizure disorders, migraine headaches) requiring treatment at the time of the experiment; pregnancy; major medical disorders that could affect cerebral metabolism, such as diabetes or thyroid disorders; current risk of suicide with a plan and intent; a Children's Depression Rating Scale Revised (CDRS-R) score >75 (extremely ill) or major depressive disorder with psychotic features; ferromagnetic metal implants or devices (e.g., electronic implants, infusion pumps, aneurysm clips, metal fragments, metal prostheses, joints, rods, or plates); BMI ≥ 25 (overweight); and visual acuity worse than 20/35 for each eye, as determined by a Snellen close vision chart (with corrective lenses allowed).

1. **Details on non-reward trial analyses**

ANCOVA revealed that no group differences were detected for control analyses: non-rewarded neutral-word trials (*F*_(1, 44)_=0.97, *p*=.333) and non-rewarded anxiety-word trials (*F*_(1, 44)_=0.39, *p*=.536).

Between-group searchlight analyses were also non-significant. However, within-groups, for the neutral-word non-rewarded trials, right dlPFC and left basolateral amygdala were observed in controls but not AN. For the anxiety-word non-rewarded trials, significant right basolateral amygdala RS was detected in AN but not controls, while clusters in the left basolateral amygdala, right NAcc, and left mOFC were significant in controls but not AN.

1. **Details on exploratory anxiety-word post-reward trials analysis**

As research [1] has shown how a positive state (e.g., positive affect after acute exercise) is associated with reductions in state anxiety, we conducted an exploratory analysis, testing if a subsequent anxiety state is influenced by a preceding reward receipt, as we reasoned that being in a rewarded state could engage overlapping anxiety and reward circuitry, thereby diminishing anxiety responsiveness. Thus, our exploratory hypothesis was that AN participant's response to anxiety provocation in anxiety regions for trials after reward receipt will be more consistent than controls.

The anxiety ROI mask used for this analysis comprised the same areas as in our previous studies [2, 3], with the exception of the centromedial amygdala nuclei instead of the entire amygdala. The other areas included the anterior cingulate cortex, insula, medial prefrontal cortex, ventral tegmental area, and bed nucleus of the stria terminalis.

ANCOVA found no significant group differences in representational similarity in the anxiety ROI mask for anxiety-word trials post-reward (*F* _(1, 44)_ = 0.05, *p* = .822). Between-group searchlight analyses did not reveal any significant differences between groups, and MANOVA did not find that any one region contributed more strongly to results. Within-group searchlights found significant clusters of representational similarity for the AN group which had much larger spatial extents than controls in the right centromedial amygdala, and anterior portions of the left insula (Figure S2, Table S3).

This exploratory analysis examined whether anxiety-word post-reward trial RS was higher in AN than controls. Although no group differences emerged, within-group searchlight analyses revealed significant RS in the right centromedial amygdala and left anterior insula in AN, with a larger spatial extent and magnitude compared to controls. The anterior insula, involved in anxiety and fear regulation[4], may play a crucial role in eating disorders and is associated with structural and functional differences compared to controls [5-7]. The centromedial amygdala is highly sensitive to negative emotional stimuli [8], and amygdala hyperreactivity to a range of stimuli [9], along with altered nuclei volume in AN [10, 11] have been reported, although these studies focused on the amygdala broadly rather than the centromedial nuclei specifically. These findings suggest consistent engagement of the anterior insula and centromedial amygdala in AN during anxiety word processing. However, as with our analysis testing if anxiety-induced states influence reward receipt RS, conclusions about the influence of prior reward receipt on these patterns are difficult to infer. From our results, there is some preliminary evidence that anxiety-word processing may differ in terms of which regions contribute more strongly to representational similarity in AN, but determining if this is due to prior reward receipt or just differences in processing anxiety stimuli may benefit from a transfer learning approach in future research.

| **Table S1** Analysis of covariance for neutral-word rewarded trials RSA with PDS as a covariate | | | | | | |
| --- | --- | --- | --- | --- | --- | --- |
| **Source** | **SS** | **df** | **MS** | **F** | ***p*** | **η² (partial)** |
| Corrected Model | 0.51 | 2 | 0.25 | 5.95 | .005 | .213 |
| Intercept | 118.23 | 1 | 118.23 | 2780.03 | < .001 | .984 |
| PDS | 0.03 | 1 | 0.03 | 0.60 | .444 | .013 |
| Group | 0.27 | 1 | 0.27 | 6.44 | .015 | .128 |
| Error | 1.87 | 44 | 0.04 |  |  |  |
| Total | 122.09 | 47 |  |  |  |  |
| Corrected Total | 2.38 | 46 |  |  |  |  |

Note. R^2^ = .213 (Adjust R^2^ = .177).

**Table S2** Searchlight analysis within-group one-sample t-test results for anxiety-word non-rewarded trials

| **Region** | **Size (voxels)** | **T max** | **Cluster #** | **MNI Coordinates** | | |
| --- | --- | --- | --- | --- | --- | --- |
|  |  |  |  | ***X*** | ***Y*** | ***Z*** |
| **Control Group** | |  |  |  |  |  |
| **Supplementary Motor Area** | 2471 | 9.44 | 7 | -4 | 8 | 46 |
| **L vlPFC/dlPFC** | 646 | 7.09 | 6 | -52 | 30 | 6 |
| **R mOFC** | 83 | 8.20 | 5 | 20 | 28 | -22 |
| **L Basolateral Amygdala** | 46 | 7.42 | 4 | -24 | -8 | -26 |
| **L mOFC** | 6 | 7.42 | 3 | -14 | 20 | -20 |
| **R NAcc** | 2 | 7.83 | 2 | 12 | 6 | -10 |
| **AN Group** | |  |  |  |  |  |
| **Supplementary Motor Area** | 2646 | 10.20 | 7 | 0 | 18 | 44 |
| **L vlPFC/dlPFC** | 249 | 7.21 | 6 | -50 | 22 | 4 |
| **L dlPFC** | 78 | 5.93 | 5 | -38 | 34 | 34 |
| **R Basolateral Amygdala** | 19 | 7.43 | 4 | 20 | -2 | -26 |
| **R mOFC** | 18 | 7.15 | 3 | 18 | 30 | -24 |
| **L dlPFC** | 3 | 5.38 | 2 | -46 | 12 | 10 |

Note. L = left hemisphere, R = right hemisphere, vlPFC = ventrolateral prefrontal cortex, dlPFC = dorsolateral prefrontal cortex, mOFC = medial orbitofrontal cortex, NAcc = nucleus accumbens, MNI = Montreal Neurological Institute, AN = anorexia nervosa

**Table S3** Searchlight analysis within-group one-sample t-test results for neutral-word non-rewarded trials

| **Region** | **Size (voxels)** | **T max** | **Cluster #** | **MNI Coordinates** | | |
| --- | --- | --- | --- | --- | --- | --- |
|  |  |  |  | ***X*** | ***Y*** | ***Z*** |
| **Control Group** | |  |  |  |  |  |
| **Supplementary Motor Area** | 2597 | 8.89 | 7 | 0 | 16 | 44 |
| **L vlPFC** | 376 | 6.39 | 6 | -50 | 32 | 6 |
| **L dlPFC** | 172 | 6.15 | 5 | -42 | 26 | 32 |
| **R mOFC** | 152 | 6.86 | 4 | 14 | 36 | -24 |
| **L mOFC** | 112 | 6.81 | 3 | -14 | 36 | 22 |
| **L Basolateral Amygdala** | 44 | 7.03 | 2 | -24 | -8 | -24 |
| **R dlPFC** | 21 | 7.13 | 1 | 38 | 34 | 36 |
| **AN Group** | |  |  |  |  |  |
| **Supplementary Motor Area** | 2180 | 8.94 | 6 | -4 | 6 | 46 |
| **L vlPFC** | 198 | 6.91 | 5 | -52 | 24 | 2 |
| **L mOFC** | 145 | 7.82 | 4 | -16 | 16 | -18 |
| **L dlPFC** | 132 | 7.41 | 3 | -46 | 24 | 32 |
| **R mOFC** | 92 | 6.09 | 2 | 22 | 32 | -18 |
| **R Basolateral Amygdala** | 1 | 6.09 | 1 | 28 | -6 | 22 |

Note. L = left hemisphere, R = right hemisphere, vlPFC = ventrolateral prefrontal cortex, dlPFC = dorsolateral prefrontal cortex, mOFC = medial orbitofrontal cortex, NAcc = nucleus accumbens, MNI = Montreal Neurological Institute, AN = anorexia nervosa

**Table S4** **Searchlight analysis within-group one-sample *t* – test for anxiety-word post-rewarded trials for AN and controls**

| **Region** | **Size (voxels)** | **T max** | **Cluster #** | **MNI Coordinates** | | |
| --- | --- | --- | --- | --- | --- | --- |
|  |  |  |  | ***X*** | ***Y*** | ***Z*** |
| **Control Group** | |  |  |  |  |  |
| **R ACC** | 1364 | 7.27 | 7 | 4 | 36 | -6 |
| **R Insula** | 196 | 7.17 | 6 | 40 | -4 | 6 |
| **L PFC** | 122 | 5.42 | 5 | -4 | 44 | -20 |
| **L Insula** | 84 | 6.19 | 4 | -40 | -6 | -10 |
| **R Insula** | 36 | 5.87 | 3 | 36 | 12 | -14 |
| **R Centromedial Amygdala** | 2 | 5.93 | 2 | 28 | -4 | -12 |
| **R Centromedial Amygdala** | 2 | 5.80 | 1 | 28 | -6 | -8 |
| **AN Group** | |  |  |  |  |  |
| **R ACC** | 1387 | 8.24 | 7 | -6 | 38 | -2 |
| **L Insula** | 385 | 8.42 | 6 | -38 | -2 | -12 |
| **R Insula** | 360 | 7.51 | 5 | 42 | 4 | -10 |
| **L PFC** | 149 | 5.47 | 4 | -4 | 38 | -18 |
| **R Centromedial Amygdala** | 23 | 6.76 | 3 | 22 | -10 | -6 |
| **L PFC** | 11 | 5.68 | 2 | -2 | 54 | -18 |
| **R Centromedial Amygdala** | 3 | 6.00 | 1 | 28 | -12 | -6 |
| *AN* anorexia nervosa, *L* left hemisphere, *R* right hemisphere, *ACC* anterior cingulate cortex, *PFC* prefrontal cortex, *MNI* Montreal Neurological Institute | | | | | | |


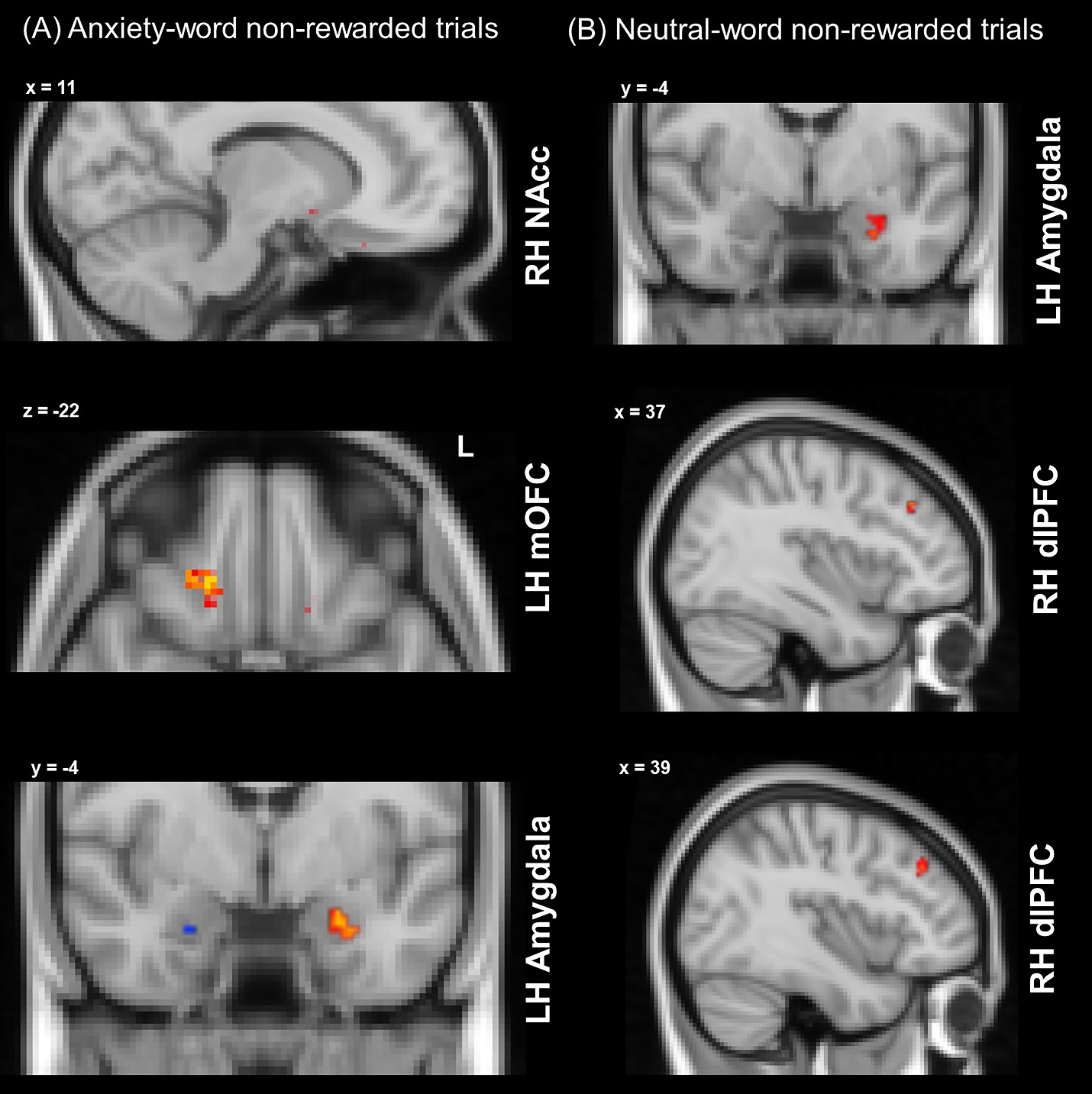


*Figure S1.* Within-group one-sample t-tests for the (A) anxiety-word non-rewarded trials representational similarity from the searchlight analysis within the reward mask, and (B), neutral-word non-rewarded trials after reward receipt representational similarity from the searchlight analysis within the anxiety mask. Within-group results are overlaid: red/yellow clusters depict significant areas in controls, while blue clusters are significant in AN (*p* < .05, corrected).

*
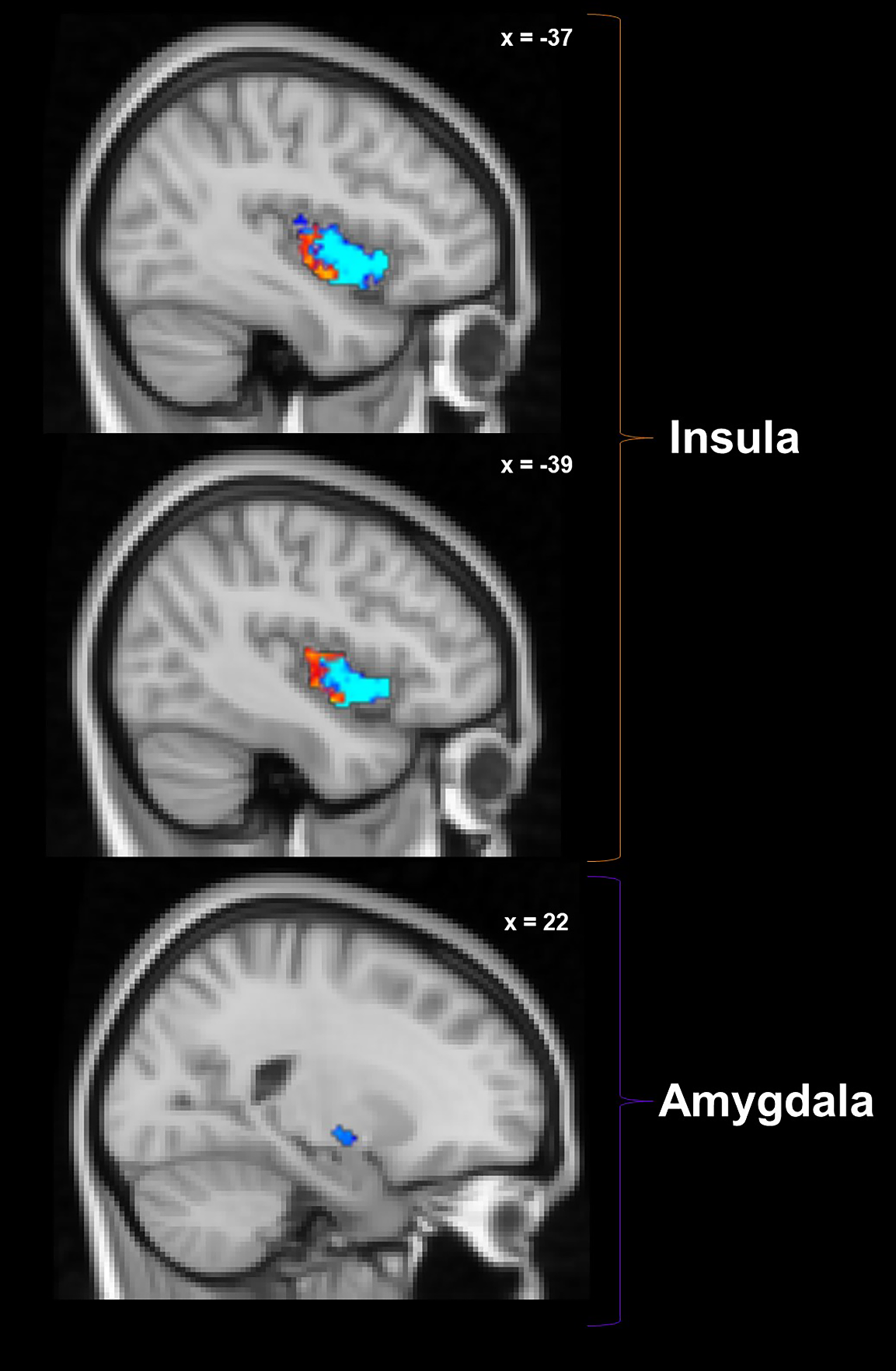
*

*Figure S2.* Within-group one-sample t-tests for the anxiety-word after rewarded trials representational similarity from the searchlight analysis within the anxiety mask. Within-group results are overlaid: red/yellow clusters depict significant areas in controls, while blue clusters are significant in AN (*p* < .05, corrected).

**References**

1. Knapen, J., et al., *State anxiety and subjective well-being responses to acute bouts of aerobic exercise in patients with depressive and anxiety disorders.* British Journal of Sports Medicine, 2009. **43**(10): p. 756.

2. Seiger, R., et al., *Neural representations of anxiety in adolescents with anorexia nervosa: a multivariate approach.* Transl Psychiatry, 2023. **13**(1): p. 283.

3. Tadayonnejad, R., et al., *Mesolimbic Neurobehavioral Mechanisms of Reward Motivation in Anorexia Nervosa: A Multimodal Imaging Study.* Frontiers in Psychiatry, 2022. **13**.

4. Shi, T., et al., *Role of the anterior agranular insular cortex in the modulation of fear and anxiety.* Brain Research Bulletin, 2020. **155**: p. 174-183.

5. Frank, G.K., et al., *Alterations in brain structures related to taste reward circuitry in ill and recovered anorexia nervosa and in bulimia nervosa.* Am J Psychiatry, 2013. **170**(10): p. 1152-60.

6. Kim, K.R., et al., *Functional and effective connectivity of anterior insula in anorexia nervosa and bulimia nervosa.* Neuroscience Letters, 2012. **521**(2): p. 152-157.

7. Oberndorfer, T.A., et al., *Altered insula response to sweet taste processing after recovery from anorexia and bulimia nervosa.* Am J Psychiatry, 2013. **170**(10): p. 1143-51.

8. Hrybouski, S., et al., *Amygdala subnuclei response and connectivity during emotional processing.* NeuroImage, 2016. **133**: p. 98-110.

9. Sharma, A., et al., *Common Dimensional Reward Deficits Across Mood and Psychotic Disorders: A Connectome-Wide Association Study.* American Journal of Psychiatry, 2017. **174**(7): p. 657-666.

10. Burkert, N.T., et al., *Body image disturbances, fear and associations with the amygdala in anorexia nervosa.* Wien Klin Wochenschr, 2019. **131**(3-4): p. 61-67.

11. Wronski, M.-L., et al., *Dynamic Amygdala Nuclei Alterations in Relation to Weight Status in Anorexia Nervosa Are Mediated by Leptin.* Journal of the American Academy of Child & Adolescent Psychiatry, 2024. **63**(6): p. 624-639.
